# Strategies for providing healthcare services to street-dwellers in Dhaka city: Evidence from an operations research

**DOI:** 10.1186/1478-4505-10-19

**Published:** 2012-06-13

**Authors:** Jasim Uddin, Tracey P Koehlmoos, Nirod C Saha, Ziaul Islam, Iqbal A Khan, MA Quaiyum

**Affiliations:** 1 International Centre for Diarrhoeal Disease Research, Bangladesh (icddr,b),Mohakhali, Dhaka 1222, Bangladesh, GPO Box 128, Dhaka, 1000, Bangladesh

**Keywords:** Street-dwellers, Healthcare, Health services, Static clinic, Satellite clinics, Urban health services, Bangladesh

## Abstract

**Background:**

In almost every major urban city, thousands of people live in overcrowded slums, streets, or other public places without any health services. Bangladesh has experienced one of the highest rates of urban population growth in the last three decades compared to the national population growth rate. The numbers of the urban poor and street-dwellers are likely to increase at least in proportion to the overall population growth of the country. The street-dwellers in Bangladesh are extremely vulnerable in terms of their health needs and healthcare-seeking behaviours. In Bangladesh, there is no health service-delivery mechanism targeting this marginalized group of people. This study, therefore, assessed the effectiveness of two models to provide primary healthcare (PHC) services to street-dwellers.

**Methods:**

This study of experimental pre-post design tested two models, such as static clinic and satellite clinics, for providing PHC services to street-dwellers in the evening through paramedics in Dhaka city during May 2009-April 2010. Both quantitative and qualitative techniques were used for collecting data. Data were analyzed comparing before and after the implementation of the clinics for the assessment of selected health and family-planning indicators using the statistical *t*-test. Services received from the model l and model 2 clinics were also compared by calculating the absolute difference to determine the relative effectiveness of one model over another.

**Results:**

The use of healthcare services by the street-dwellers increased at endline compared to baseline in both the model clinic areas, and the difference was highly significant (*p* < 0.001). Institutional delivery among the female street-dwellers increased at endline compared to baseline in both the clinic areas. The use of family-planning methods among females also significantly (*p* < 0.001) increased at endline compared to baseline in both the areas.

**Conclusions:**

As the findings of the study showed the promise of this approach, the strategies could be implemented in all other cities of Bangladesh and in other countries which encounter similar problems.

## Background

In almost every major city, thousands of people live in overcrowded slums, streets, or other public places without any health services [1]. Bangladesh has experienced one of the highest rates (>6% per year) of urban population growth in the last three decades compared to the national population growth rate of about 1.5% per year [1-4]. The numbers of the urban poor and street-dwellers are likely to increase at least in proportion to the overall population growth [2,4-6].

Street-dwellers are defined as people who sleep on streets, railway terminals and platforms, bus stations, parks and open spaces, religious centres, construction sites, around graveyards, and other public places [2,7,8]. They are extremely vulnerable in terms of their health needs and healthcare-seeking behaviours. Their health problems are broad and multidimensional, contributing to excess mortality [9-14]. Rates of acute and chronic medical illnesses among them are high [15-22] but they have poor access to healthcare [23-27]. A study in Dhaka city found that 72% of female and 48% of male street-dwellers were sick at the time of data-collection [28]. The female street-dwellers were mostly affected by reproductive health problems, such as vaginal discharge, lower abdominal pain, genital itching/burning, and others, such as mass in the lower abdomen, irregular period, and prolapse. The common general illnesses reported by them included diseases of the respiratory system (cold/cough/fever/asthma), diseases of the digestive system (gastric, diarrhoea), severe pain (headache/chest), and scabies. The findings of the study showed that about half of female and one-third of male street-dwellers did not seek healthcare services during their illness. Only 28% of pregnant women sought antenatal care (ANC). Of those who sought healthcare services, more than half of females and two-thirds of males bought medicines from drug-sellers at the nearest pharmacy. A few street-dwellers who visited healthcare facilities encountered problems, such as denial of treatment by providers due to their financial insolvency, neglect of service providers for services, and an inappropriate service-delivery time for them [28]. The findings of the study revealed that, although there are mechanisms to provide primary healthcare services (PHC) to other groups of people, such as slum-dwellers and people living in hard-to-reach areas, by the Ministry of Health and Family Welfare (MoHFW) and non-government organizations (NGOs), there is no health service-delivery mechanism targeting this marginalized group of people in Bangladesh. The study findings also showed that although there is no mechanism for providing services to this marginalized group of people, they used to visit to the clinics of MoHFW and NGOs when they become severely ill, and receive treatment with negligence [28,29]. The results of an earlier study also suggest that street-dwellers cannot access conventional healthcare services due to the financial and time constraints linked to their livelihoods [30].

In exploring how to make healthcare services more accessible to street-dwellers, the specific requirements that they described were longer, more flexible opening hours, free or low-cost services, and quality services provided by paramedics [30]. The existing service-delivery system is not convenient for them because the usual service hours do not match their free time due to their work schedule. Although there are pharmacies where they can buy medicines, the drug-sellers are not medically trained and are not able to provide quality advice or treatment. Consequently, their health needs remain largely unmet through the existing healthcare providers [30]. In many countries, initiatives have been taken to address the health needs of street-dwellers [31,32]. However, Uddin et al. recommended that both mobile and static clinics might be feasible to provide healthcare services to urban street-dwellers in Bangladesh [30].

Although the Health and Population Sector Programme of Bangladesh designed programmes to ensure equitable essential services to all, this marginalized group of people was not, however, targeted. This warrants that the MoHFW examines its role to focus its future programmes to meet the needs of this extreme vulnerable group of people. The present study, therefore, developed strategies for providing PHC services to street-dwellers in Dhaka city. The strategies included static and satellite clinics. The purpose of this study was to test the strategies and assess its effectiveness in improving the use of healthcare services by street-dwellers in Dhaka city.

## Methods

### Study design and study sites

This study of experimental pre-post design was conducted in two of 11 selected areas of Dhaka city during May 2009-April 2010. A pre-post test design was chosen to collect data on study participants’ level of performance before the intervention took place (pre), and that we collected same data on where study participants were after the intervention took place (post). This design is the best way to be sure that the interventions had a causal effect. However, the study did not include control group due to fund constraints. The study areas included five major entry points and six particular locations with major concentrations of street-dwellers [28]. The entry points were defined as the locations where people came from rural areas and landed there to stay in the city. The entry points included railway stations, bus stations, and launch terminals. The concentrated areas included parks and open spaces, religious centres, construction sites, around graveyards, and other public places where street dwellers mostly stay. Two of the 11 areas—Kamlapur (entry point) and Karwan Bazar (concentrated area)—were purposively selected for the present study. The study covered the two-kilometre radius area of each site. Two model clinics—model 1 (static clinic) and model 2 (satellite clinics)—were started in Karwan Bazar and Kamlapur respectively in 2009.

### Implementation

#### Model 1: Static clinic

Previous discussions on behaviours of street-dwellers and anecdotal evidence considered that they are a highly-mobile population so that designing any long-term programmes for them would not be effective. However, a recent study reported that half of street-dwellers stay in the same place for three years and more [28]. The findings also showed that a good percentage of them stay in the same place for 10 years and more. The findings of another study showed that 54% of street-dwellers were inhabited in one place for the past four years (33). The findings also showed that 21% of street-dwellers resided in a same location for 15 years and more. The studies, therefore, recommended the establishment of static clinics for providing health services to street-dwellers. Based on these findings, the present study established one static clinic in one selected area of Dhaka city for providing services to them. The concerned zonal office of the Dhaka City Corporation (DCC) allocated a room for this purpose. Two paramedics—one female and one male—were engaged in providing services from the static clinic. A package of PHC services was provided from the clinic to street-dwellers (Table 1). The PHC services were provided from the clinic in the evening (i.e. from 6:30 pm to 10:00 pm) thrice a week.

#### Model 2: Satellite clinics

As stated, there is no service-delivery system targeting the needs of street-dwellers. Although there are other healthcare facilities surrounding the concentrated areas of street-dwellers, all are, however, involved in providing services during the day time when the street-dwellers remain outside for searching of food for them. In view of this, the project organized evening satellite clinics. The mobile satellite clinics were organized in the railway station and stadium complex. In absence of any room to provide services, a makeshift clinic was used.

**Table 1:** Services provided from clinics

	
**Treatment/services provided on**	**Name of diseases and services offered**
**General health**	**Gastritis, hypertension, anaemia, pain (headache and others), common cold and cough, fever, asthma, scabies, infection (any type), injury (any type), diarrhoea, dysentery, and health education**
**Reproductive and maternal health**	**Sexually transmitted infections (STIs)/reproductive tract infections (RTIs), ANC, postnatal care (PNC)**
**Family-planning methods**	**Condom, pill, and injection**
**Child health**	**Acute respiratory infection (ARI), pneumonia, diarrhoea, dysentery, common cold and cough, fever, scabies, helminthiasis, infection (any type), and injury (any type)**
**Expanded Programme on Immunization (EPI)**	**BCG, pentavalent (DPT, hepatitis B, and Hib), OPV, measles, and tetanus toxoid**
**Referral to government and NGO facilities**	**Delivery, semi-permanent and permane family-planning methods (Norplant, intrauterine device, tubectomy, and vasectomy)**

The paramedics who worked in the model 1 clinic also provided services in the model 2 clinic areas in alternate evenings (from 6:30 pm to 10:00 pm) as the street-dwellers return from their work at this time. The same package of PHC services that were provided from the static clinic was as well provided from the satellite clinics for the street-dwellers.

The package of services that were provided from both static and satellite clinics are presented in Table 1.

A system of referral linkage was established with the nearer health facilities, managed by the Government and NGOs, to refer patients from both static and satellite clinics. The paramedics referred cases to these referral points, and a Field Research Officer (FRO) followed up the cases to know what happened after referral.

A decorated rickshaw van was used for both the types of clinics for carrying the clinic staff and logistics, for publicity among the target people, and for carrying patients from the static and satellite clinics to the referral points, if necessary.

The main components of the interventions were same, except the type of clinics (Table 2).

**Table 2:** Components of interventions

	
**Static clinic**	**Satellite clinics**
**Organized in a room provided by Dhaka City Corporation**	**Organized in public utilities. Since no room was available, a makeshift clinic was used in the satellite clinics**
**Services provided in every alternate day (three days a week)**	**Services provided in every alternate day (two days a week)**
**Clinic hours: 6:30 pm to 10:00 pm**	**Clinic hours: 6:30 pm to 10:00 pm**
**Two paramedics—one female and one male—provided services from the static clinic**	**Same paramedics of the static clinic provided services from the satellite clinics**
**A package of PHC services (the list provided above) was provided to the street-dwellers**	**The same package of PHC services was provided to the street-dwellers**
**A patient card was provided to the clients so that it became easy to keep track in subsequent visits**	**The same card was provided to the clients so that it became easy to keep track in subsequent visits**
**A system of referral linkage was instituted from the clinic to the nearer health facilities managed by the Government and NGOs**	**A system of referral linkage was instituted from the clinics to the nearer health facilities managed by the Government and NGOs**
**A decorated rickshaw van was used for carrying the clinic staff and logistics, for publicity among the target people, and for carrying patients from the static clinic to the referral points, if necessary**	**A decorated rickshaw van was also used for carrying the clinic staff and logistics, for publicity among the target people, and for carrying patients from the satellite clinics to the referral points, if necessary**

To ensure the quality of services, the paramedics were trained by the experts from the primary healthcare programme of the MoHFW following the essential services-delivery protocol. The study investigators used a standard checklist in both the models for monitoring and supervising activities of the clinics and service providers. As the same service providers were involved in the satellite and static clinics, it was possible to ensure the similar quality of services in both the types of clinics. As the street-dwellers are extremely vulnerable groups, special focus was given during training of the service providers so that they become non-judgemental in providing services to street-dwellers. The service providers were also oriented towards attitudes and motivational aspects for dealing with street-dwellers. Both classroom and field training was provided to the paramedics. The main components of classroom and field training included: health education, diagnosis and treatment of general health problems, including infection prevention, reproductive health, maternal health, child health, referral system, and communication and behavioural aspects. After the completion of a 15-day classroom training, practical training at the NGO and government clinics were provided to the paramedics. They worked practically with doctors of the NGO and government healthcare facilities for another 15 days before they began to provide services from the clinics.

The drug-sellers of local pharmacies were used for informing street-dwellers about the availability of services for them from the model 1 and model 2 clinics. Other public healthcare providers in the areas were also informed. Names and addresses of the clinics and types of services available in the static and satellite clinics were provided to the drug-sellers and other providers. They were motivated to inform the street-dwellers about the availability of services in the model clinics and also to motivate them to visit the clinics for services.

The services were provided to those street-dwellers who slept for the last one week in the study areas. A patient card was provided to all the clients to keep their track in subsequent visits. The paramedics ascertained the duration of their stay in the area by asking question about this at the time of their first visit before providing the card. Health services were not provided to any street-dwellers who did not fall in the inclusion criterion. They were considered either too mobile or they may leave the street anytime. All the agegroups of the street-dwellers in the study areas received health services from both static and satellite clinics, although for the purpose of evaluation the study subjects must be aged 15 years and above.

The icddr,b, in collaboration with the Urban Health Programme of the MoHFW, DCC, Urban Primary Health Care Project of the Ministry of Local Government, Rural Development & Co-operatives-LGRD, and NGOs, implemented the interventions. For monitoring the project activities, a project management committee, headed by Director, PHC of the MoHFW, was formed. The committee met quarterly, reviewed progress of the activities, and provided necessary feedback and guidelines to the research team.

### Evaluation

#### Study subjects

To obtain information on reproductive health, maternal health, and family planning and to receive services from the clinics, the following criteria for inclusion as study subjects were set: street-dwellers must be ever-married females and males aged 15 years and above, living within the two-kilometre radius of the study locations; and were sleeping in the area for at least one week before data-collection.

#### Sampling

We had drawn two samples of adults (male and female) from the street-dwellers who slept in the last week within the area of the two-kilometre radius of the model clinics. To estimate a 10% increase in the use of services by the street- dwellers from the existing 34% from the government/non-government facilities for any ailment, with 95% confidence interval, and 80% power, the required sample-size was 200 ever-married females and 200 ever-married males aged 15+ years from each study area. In total, 800—400 females and 400 males—were interviewed at baseline and endline from the two study areas.

Since the street-dwellers remain out of their sleeping places for the whole day in searching of food for them, data were collected at night when the street-dwellers took preparation to sleep. The two study areas were divided into number of blocks based on different points/locations. Total sample size of each study area was also divided as per number of blocks and population size of each block. The interviewers visited the centre of a block and spun a bottle to randomly select a direction. The interviewers were then went to the border of the selected direction of a block and interviewed all eligible persons following the clockwise direction till the completion of interviews of the target population from each block. A group of female and male interviewers conducted the interviews.

#### Data-collection

The mixed method approach, combining both quantitative and qualitative techniques, was used for data-collection. The community survey and qualitative components (in-depth interviews with study subjects and healthcare providers) were carried out.

##### Sample Survey

A team of experienced male and female interviewers conducted the survey of adult street-dwellers and collected data using a structured questionnaire, supervised by the researchers. The male interviewers interviewed the male street-dwellers, and the female interviewers interviewed the female street-dwellers. The questionnaire was developed and pretested before data-collection. Since the morbidity and healthcare indicators were of concerns, the interviewers were trained with special focus on these issues. Both classroom and field training was imparted to them. The interviewers asked questions to street-dwellers about morbidity and use of healthcare services in simple languages so that they could understand and respond easily. Information was collected on the sociodemographic characteristics of street-dwellers, morbidity, use of healthcare services, and the type of facility visited for treatment.

Qualitative data were collected to supplement the quantitative data. Experienced Field Research Assistants (FRAs) collected qualitative data.

##### In-depth interviews with street-dwellers

In-depth interviews were conducted with ever-married female and male street-dwellers. The field research assistants (FRAs) used the in-depth interview guidelines during interview. They were trained on the guidelines before data-collection. In total, 12 street-dwellers were interviewed from each sex. Street-dwellers who were sick during the last two weeks were selected for interview. They were selected in such a way that it represents young adults, currently married, and pregnant mothers. Data were collected on the frequency of use of healthcare facilities for the treatment of their diseases, awareness about old and new systems, use of the model 1 and 2 clinics, source of information about the clinics, barriers they faced in receiving services, perceptions about the new and old systems, and also about their perceptions of services received (referral, especially concerning free services).

##### In-depth interviews with healthcare providers

In-depth interviews were also conducted with the service providers. A list of the service providers in the study areas was available from an earlier study [28]. It was updated for the selection of respondents for interview. The FRAs used separate guidelines in interviewing them. Twelve randomly-selected service providers, including paramedics involved in providing services in the model 1 and 2 clinics and NGOs, and drug-sellers from each study area were interviewed. Information was collected on the use of clinics by the street-dwellers, barriers faced by the providers in providing services, perceptions about the systems, and their suggestions for improving the strategies.

### Analysis of data

Data were analyzed to assess the effectiveness of the interventions in improving the use of healthcare services among street-dwellers in Dhaka city. The quantitative data from the survey were entered into visual Basics/FoxPro and analyzed with the SPSS PC + software. The quality of data was maintained through extensive supervision and checking to ensure that errors are minimized. Quantitative data were analyzed by comparing before and after the implementation of the clinics for the assessment of selected health and family-planning indicators using statistical the *t*-test. The comparison of services received from the model l and 2 clinics was also done by calculating the absolute difference to determine the relative effectiveness of one model over another.

Qualitative data collected through in-depth interviews were transcribed, translated into English, and analyzed using content analysis. Analysis of qualitative data began with the first field activities and was refined as the study proceeded. The data-analysis processes followed a sequence of interrelated steps, such as reading, coding, displaying, reducing, and interpreting. At first, the transcripts were read carefully, and then coding the data was done. Reading and coding were initiated while the data were collected. The data-display and reduction processes were conducted at desk once all the data had been collected. Even during data display and reduction, the investigators reviewed the earlier steps to refine codes and created texts and revised some aspects of the analysis.

### Ethical approval

The Research Review Committee and the Ethical Review Committee of the icddr,b approved the study. Respondents were interviewed after obtaining written consent from them. In the case of illiterate participants, thumb impression was obtained in the consent form. Efforts were made (read-out a consent form containing the purpose of the study, why their participation in the study was necessary, privacy, and other ethical issues) to ensure that all the respondents were properly informed about the study and they thoroughly understood what their involvement in the study is. Participation was voluntary. The participants were free to refuse participation in the study, and they were ensured that they would have no adverse consequences.

## Results

### Sociodemographic information

A total of 804 (403 female and 401 male) street dwellers were interviewed. There was no refusal case. Overall, 8% (n = 403) and 13% (n = 401) of the female respondents at baseline and endline respectively were adolescents. About half of both female and male street-dwellers were aged 20–39 years. Of the female respondents, about half at baseline and one-third at endline were widowed, abandoned, divorced, or separated. More than 75% of the females and males had never gone to any school. Status of never gone to school was higher among female street dwellers than that of the male street-dwellers both at baseline and endline. The main occupations of the females were vegetable- and waste-picking and selling, begging, day labour, and domestic help while the main occupations of the male street-dwellers were day labour, rickshaw/van-pulling, begging, and small business. The findings revealed that about 95% female and male street-dwellers had been staying in the street for more than a year. About one-third of the street-dwellers had stayed in the street for 3–10 years. The findings also revealed that approximately one-third had stayed in the street for more than 20 years.

### Morbidity among street-dwellers

Current sickness among the female street-dwellers at endline decreased in both model 1 and model 2 clinic areas, and the difference of decrease in sickness was highly significant (*p* < 0.001) (Table 3). The absolute difference of decrease in current sickness among them in the model 1 and 2 clinics was 35% (relative difference 42%) and 15% (relative difference 18%) respectively.

**Table 3:** Status of morbidity among street-dwellers

**Morbidity**	**Females**					
	**Model 1**			**Model 2**		
	**Baseline (n = 203) No. (%)**	**Endline (n = 200) No. (%)**	**% of difference**	**Baseline (n = 200) No. (%)**	**Endline (n = 201) No. (%)**	**% of difference**
Currently sick	168 (83)	96 (48)**	35	166 (83)	137 (68)**	15
Reported illness during the past two weeks (if not currently sick)	18 (51)	50 (48)	3	14 (40)	20 (31)	9
Morbidity	**Males**					
	**Model 1**			**Model 2**		
	**Baseline (n = 201) No. (%)**	**Endline (n = 200) No. (%)**	**% of difference**	**Baseline (n = 200) No. (%)**	**Endline (n = 201) No. (%)**	**% of difference**
Currently sick	179 (89)	118 (59)**	30	160 (80)	143 (71)	9
Reported illness during the past two weeks (if not currently sick)	14 (64)	49 (60)	4	16 (40)	20 (35)	5

**p < 0.001.

Current sickness among the male street-dwellers at endline also decreased significantly in the model clinic 1 area compared to that at baseline. The absolute decrease in current sickness among them in the model 1 and 2 clinics was 30% (relative difference 34%) and 9% (relative difference 11%) respectively.

The table shows that, although there was no significant difference, sickness among the female and male street-dwellers during the past two weeks decreased at endline compared to baseline.

The street-dwellers were affected mainly by reproductive and general health problems. The reproductive health problems included lower abdominal pain, vaginal/urethral discharge, and genital itching/burning. The major general health problems reported by them included diseases of the respiratory system, weakness, severe headache, chest pain, and diseases of the digestive system.

### Use of healthcare services

The female street-dwellers used healthcare services at a higher rate after the implementation of the clinics compared to baseline (Table 4), and the difference was highly significant (*p* < 0.001). The table shows that the absolute difference of increase in the use of healthcare services by them between baseline and endline was 56% (relative difference 140%) and 31% (relative difference 100%) respectively.

**Table 4:** Use of healthcare services among street-dwellers

**Use of healthcare services**	**Females**					
	**Model 1**			**Model 2**		
	**Baseline (n = 186) No. (%)**	**Endline (n = 146) No. (%)**	**% of difference**	**Baseline (n = 179) No. (%)**	**Endline (n = 157) No. (%)**	**% of difference**
Sought healthcare services	74 (40)	139 (96) **	56	55 (31)	97 (62)**	31
	**Males**					
	**Model 1**			**Model 2**		
	**Baseline (n = 195) No. (%)**	**Endline (n = 167) No. (%)**	**% of difference**	**Baseline (n = 176) No. (%)**	**Endline (n = 163) No. (%)**	**% of difference**
	140 (72)	165 (99)**	27	70 (40)	121(74)**	34

**p < 0.001.

The table also shows that the use of healthcare services by the male street-dwellers at endline increased significantly compared to baseline in both model 1 and 2 clinic areas. The absolute difference of increase in the use of services by them between baseline and endline was 27% (relative difference 38%) and 34% (relative difference 85%) respectively in the model 1 and 2 clinic areas.

The street-dwellers mostly bought medicines from the drug-sellers at the nearest pharmacy at baseline in both the clinic areas (Table 5). However, their use of pharmacy greatly decreased at endline. The use of model 1 and 2 clinics by the females and males was remarkably high at endline compared to that at baseline.

**Table 5:** Type of clinic/facility visited by street-dwellers for services

Type of clinic/facility visited*	**Females (%)**				**Males (%)**			
	**Model 1**		**Model 2**		**Model 1**		**Model 2**	
	**Baseline**	**Endline**	**Baseline**	**Endline**	**Baseline**	**Endline**	**Baseline**	**Endline**
	**(n = 74)**	**(n = 139)**	**(n = 109)**	**(n = 97)**	**(n = 140)**	**(n = 165)**	**(n = 122)**	**(n = 131)**
Pharmacies/drug stores	64	25	56	9	85	19	75	37
Government clinics/hospitals	28	1	14	1	19	14	15	3
Static clinic (Model 1)	0	71	0	0	0	93	0	0
Satellite clinics (Model 2)	0	0	0	79	0	0	0	77
Quacks/doctors/streetvendors	5	0	6	0	12	0	3	0
NGO clinics	4	7	29	6	12	2	5	1
Homeopath and ayurvedic pharmacy	13	2	9	6	9	5	6	2
Others	1	2	5	5	4	3	11	4

*Multiple responses.

The table also shows that the use of government and NGO clinics for healthcare services by the street-dwellers decreased at endline compared to baseline. The availability of services from the static and satellite clinics at their sleeping places/nearby sleeping places and at a convenient time (evening) may be the reasons for not seeking services from the government and NGO clinics.

### Maternal and reproductive health

The street-women who were not currently pregnant were asked if they had given births during the last 12 months preceding data-collection. The findings showed that 18% and 17% of the mothers at baseline and endline respectively gave births in the model 1 clinic area (Table 6). In the model 2 clinic areas, 11% and 13% of the mothers gave births at baseline and endline respectively. The table shows that 65% of deliveries were conducted at the street in the model 1 clinic area before the implementation of the model clinics while there was zero delivery at the street after the implementation of the clinics in the area. The rate of deliveries in the street decreased from 50% to 20% after the implementation of the model 2 clinic in its areas. The number of deliveries in the public, NGO and private clinics increased after the implementation of the model clinics compared to before their implementation, except the public facilities in the model 2 clinic areas. The table also showed that the rate of deliveries assisted by relatives came down from 59% at baseline to 0% at endline in the model 1 clinic area. The rate of deliveries assisted by relatives decreased from 40% at baseline to 24% at endline in the model 2 clinic areas. The number of deliveries conducted by the trained personnel, such as nurses/midwives and MBBS doctors, increased at endline compared to baseline in both the model clinic areas.

**Table 6:** Status of delivery and delivery care among street-women who gave births during the previous 12 months

**Delivery and delivery care**	**Percentage**			
	**Model 1**		**Model 2**	
	**Baseline**	**Endline**	**Baseline**	**Endline**
	**(n = 194)**	**(n = 188)**	**(n = 190)**	**(n = 190)**
Gave births during the past 12 months	18	17	11	13
Place of delivery				
Street	65	0	50	20
At clinic (public and NGO)	6	48	10	40
At home (village home or rented home in slums at that time)	29	52	40	40
Delivery assisted by				
Trained personnel (MBBS doctor, nurse, midwife)	6	56	10	40
Relative and neighbour	59	0	40	24
Traditional birth attendant	32	44	45	36
Nobody (self)	3	0	5	0

### Use of family-planning methods among street-dwellers

The use of family-planning methods among the females significantly (*p* < 0.001) increased at endline compared to baseline in both the model clinic areas (Table 7). The table shows that absolute difference of increase in the use of family-planning methods by the females between baseline and endline as 32% (relative difference 91%) and 29% (relative difference 161%) respectively in the model 1 and 2 clinic areas. The use of family-planning methods significantly (*p* < 0.001) increased among the males also at endline compared to baseline in the model clinic 1 area. The absolute difference of increase in the use of family-planning method by the males between baseline and endline was 44% (relative difference 133%) and 11% (relative difference 37%) respectively in the model 1 and 2 clinic areas.

**Table 7:** Status of family-planning methods used by female and male street-dwellers

**Use of familyplanning methods**	**Females**					
	**Model 1**			**Model 2**		
	**Baseline**	**Endline**	**% of difference**	**Baseline**	**Endline**	**% of difference**
	**(n = 194) No. (%)**	**(n = 188) No. (%)**		**(n = 190) No. (%)**	**(n = 190) No. (%)**	
Used methods	68 (35)	126 (67)**	32	34 (18)	89 (47) **	29
Type of method used						
Temporary	20 (30)	29 (23)	7	17 (51)	43 (48)	3
Semi-permanent	38 (55)	76 (60)	5	8 (23)	31 (36)	13
Permanent	8 (12)	17 (14)	2	6 (17)	11 (12)	**-5**
Traditional	2 (3)	4 (3)	0	3 (9)	4 (4)	5
	**Males**					
	**Model 1**			**Model 2**		
	**Baseline (n = 167) No. (%)**	**Endline (n = 176) No. (%)**	**% of difference**	**Baseline (n = 165) No. (%)**	**Endline (n = 76) No. (%)**	**% of difference**
Used methods	55 (33)	136 (77)**	44	50 (30)	31 (41)	11
Type of method used						
Temporary	29 (52)	73 (54)	2	24(48)	13 (41)	7
Semi-permanent	22 (41)	53 (39)	-2	16 (32)	15 (47)	15
Permanent	4 (7)	10 (7)	0	10 (20)	3 (12)	**-8**
Traditional	0	0		1(2)	0	

**p < 0.001.

The use of temporary methods among the female street-dwellers slightly decreased at endline compared to baseline in both the model clinic areas. On the other hand, the use of semi-permanent and permanent methods among the females slightly increased at endline compared to baseline in both the model clinic areas, except permanent methods in the model clinic 2 areas. The use of temporary methods among the males slightly decreased at endline compared to baseline in the model clinic 2 areas. The use of semi-permanent and permanent methods among the males remained almost same at endline in model clinic 1 areas while it increased in model clinic 2 areas. There was a negative trend in the use of permanent methods by the females and males in the model clinic 2 areas. Also, there was not much change observed in the use of permanent methods by the females and males in the model clinic 1 area. Absence of provision of providing permanent methods from the model clinics may be the reason behind this.

### Perceptions of street-dwellers about model clinics

The survey data showed that 80% of the street-dwellers in both the clinic areas were satisfied with the services received from the model clinics. The street-dwellers who participated in in-depth interviews in the model 1 and 2 clinic areas stated that the clinics were very helpful for those who live on the street.

All the participants of both the clinic areas reported that the model clinics were different from other health facilities in the following presented in Table 8.

**Table 8:** Perceptions of street dwellers about model clinics and comparison with other health facilities

**Model clinics**	**Other clinics**
The model clinics provide services in the evening, which was very convenient for street-dwellers to get services	Other health centres open at day time when street-dwellers cannot go there as they become busy and remain out of their sleeping places in search of food for them
The staff members of the model clinics come to them and call them to visit their clinics. The providers of the model clinics respect them, listen to them, and provided services carefully	There is nobody to call them to go to clinics. The providers of the clinics avoid and discourage them to visit those clinics
There are male and female doctors for them at the model clinics, and services are provided to female patients by the female providers	In most cases, there is no provider from separate sex
Behaviour of the providers of the model clinics is very good, and they are very polite	Providers at other hospitals do not behave well with street-dwellers
The model clinics are very near to their sleeping places. No money is needed to visit the clinics	There is no other clinic/hospital near their sleeping places
There is very good discipline in the model clinics, and no need to wait for a long time	Requires much time to get treatment because other clinics/hospitals provide services to street-dwellers after providing services to other patients. The providers of that clinics neglect street- dwellers

### Perceptions of service providers about model clinics

The service providers who participated in in-depth interviews were well aware of the model clinics. They informed that, at the beginning of establishing the model clinics, the clinic organizers arranged meetings with them and informed them about the activities of the clinics, objectives of establishing the clinics, clinic schedule, and types of services offered from the clinics. All of them could tell about types of services provided from the clinics, who are the providers in the clinics, clinic schedule, and the daily patient-load in the clinics. The pharmacist informed that they used to send sick street-dwellers to the model clinics for treatment. The service providers of NGOs said, “We are also the part of the new initiative”. We provided services to the street-dwellers who were referred from the model clinics. They informed that, on average, 50 patients were visiting the model clinic daily. One paramedic from the model clinic said:

Fifty street-dwellers came to the model clinics daily for healthcare services but females came in a large number than males. The unmarried people came in a less number. The highest number of mothers of children and middle-aged women came to get treatment for them.

The service providers of NGOs and the pharmacist mentioned that the establishment of the model clinics for street-dwellers was very helpful for the target people and for them as well. They stated that the street-dwellers are extremely poor people; they do not have money for the treatment of their illnesses. The street-dwellers used to visit them before the introduction of the the model clinics and sought treatment without money. However, the street- dwellers mostly visited the model clinics after the introduction of the clinics instead of visiting the service providers, which helped them provide services to limited street-dwellers without money. The service providers also provided services to those street-dwellers who were referred from the model clinics, and they did not face any problem. One paramedic of an NGO said:

No, we did not face any problem with the model clinics. On the contrary, it was an advantage for us because when the street-dwellers come to us we usually avoid them. Despite our efforts to avoid them, they are often reluctant to leave without any medicines. As the poor people are unable to pay money for medicines, we had to give some medicines to them free of charge. Considering this circumstance, I think that the model clinics are supporting us. After establishing the clinics, we have been providing services to selected street-dwellers who are referred from the model clinics.

## Discussion

The findings showed that over the period during which the model clinics were implemented, the use of healthcare services by the street-dwellers improved significantly in both the clinic areas. Improvements were observed in the proportion of reduction in morbidity and in the use of general, reproductive and maternal healthcare services among them in the model 1 and 2 clinic areas. These improvements indicate a positive overall impact of both the model clinics.

Sickness among the female and male street-dwellers at endline decreased in both the model clinic areas, and the difference in the decrease of sickness was highly significant. Providing health education to the street-dwellers by the paramedics from both static and satellite clinics were an important component. The rate of morbidity declined possibly due to increased health awareness among the street-dwellers, which was created by providing health education to them and because of the use of healthcare services from the model clinics. Morbidity among male and female street dwellers during past two weeks remained almost same between baseline and endline. Recall bias may be a factor about this.

In general, the number of deliveries in the public and NGO clinics increased after the implementation of the model clinics compared to before their implementation. The number of deliveries conducted by the nurse/midwives and MBBS doctors increased at endline compared to baseline in the model clinic areas. The use of family-planning methods among the street dwellers increased significantly at endline compared to baseline in the model clinic areas. Thus, the findings clearly indicate the positive impacts of the model clinics in improving the health status of the street-dwellers, and the findings are consistent with those of an earlier study [33].

The use of the model clinic 1 by the street-dwellers was slightly higher compared to the model clinic 2. In general, the absolute difference in the reduction of morbidity and the increase in the use of healthcare services between baseline and endline were higher in the model 1 clinic than in the model 2 clinic. More acceptability of the static clinic among the street-dwellers than the satellite clinics may be a reason for this, which should be considered by policy makers in designing programmes for street dwellers.

Since the street-dwellers remain out of their sleeping places for the whole day in search of food, it is not possible for them to get services before evening [28]. Besides, they are unwilling to go far to get services due to cost of transportation [34]. Considering this problem, services from the model clinics were provided in the evening, and the clinics were established nearer to their sleeping places. The street-dwellers also recommended consideration of these issues while organizing services from the clinics [30]. Hence, the time and the place of service-delivery for street-dwellers are crucial.

The paramedics provided healthcare services from the model clinics. A system of referral linkage was established from these clinics to the nearer health facilities managed by the Government and NGOs. Since there was no facility to conduct delivery at the model clinics, all the delivery cases were referred to the referral centres by the paramedics. The findings revealed that the number of deliveries in the referral clinics, such as public and NGO clinics, increased at endline compared to baseline. Deliveries conducted by the nurse/midwives and MBBS doctors also increased at endline compared to baseline in both the model clinic areas. The referral centres provided services to the street-dwellers free of charge. Therefore, the role of referral centres in providing healthcare services to the street-dwellers is important.

Besides the effectiveness of the model clinics, scalability and sustainability are the key issues for any effective intervention. Although neither scalability nor sustainability was formally assessed in the present study, a number of factors, such as the increased use of clinic services by the street-dwellers, their acceptability of the clinic activities, and perceptions of the service providers suggest that the model clinics are both scalable and sustainable. The MoHFW and NGOs are already trying to develop a mechanism to provide PHC services to this group of population.

Street-dwellers in Dhaka city have limited access to effective healthcare services and are vulnerable to poor health. The existing service-delivery system is not convenient for them as the usual service hours do not match their free time. Although they may buy medicines from the existing pharmacies, the drug-sellers are not, however, medically trained and cannot provide quality advice or treatment to them. Consequently, their health needs remain largely unmet through the existing healthcare providers [30]. The question is: how the healthcare needs of this population can be met?. This is a difficult problem because the demands for healthcare services far exceed the resources available to meet them, not only for this group but for other impoverished groups [31]. However, the findings of this operations research developed strategies for providing healthcare services to this group of population, which was essentially needed in the public-health sector of this country.

In general, different contextual factors that may affect the implementation of strategies need to be considered while applying this approach beyond the study areas. In Bangladesh, the places where either mainly the Government or NGOs currently provide PHC services, the clinics can be implemented. Given the varied success of the health systems globally, it is clear that careful attention is needed to context before implementing any strategy in a particular setting [35,36].

### Limitations

There is a clear potential for confounding from other efforts to improve the use of services but there was no evidence of other interventions in the study areas during the study period. The areas used in the pre- and post-intervention surveys were the same. The fact that they were selected from the same areas, which are homogenous in terms of characteristics of the inhabitants and health services, suggests that this should not have confounded the results.

## Conclusions

The previous research suggests that street dwellers cannot access conventional health care services due to the financial and time constraints linked to their livelihoods. In exploring how to make healthcare services more accessible to street dwellers, the specific requirements that they described were longer, more flexible opening hours, free or low cost services, and quality services provided by paramedics [30]. The existing service delivery system is not convenient for street dwellers because the usual service hours do not match their free time due to their work schedule. The idea of static and satellite clinics were efficient way to increase their access to health services. The available data suggest that the model clinics had a positive impact. The findings support the need for such models to solve problems, such as absence of a service-delivery mechanism. However, such strategies will need to target specific groups of people who may have less access to health services. As the findings showed the promise of this approach, the strategies could be implemented in all other cities of Bangladesh and in other countries which encounter similar problems.

## Abbreviations

ANC: Antenatal Care; DCC: Dhaka City Corporation; ESD: Essential Service-delivery; FRO: Field Research Officer; FRA: Field Research Assistant; GIZ: German International Technical Cooperation; icddr,b: International Centre for Diarrhoeal Disease Research, Bangladesh; MoHFW: Ministry of Health and Family Welfare; MoLGRD: Ministry of Local Government, Rural Development & Co-operatives; RTI: Reproductive Tract Infection; STI: Sexually Transmitted Infection; NGO: Non-government Organization; PHC: Primary Health Care.

## Competing interests

The authors declare that they have no competing interests.

## Authors’ contributions

This article critically reflects on a research project in which the authors have been involved. All the six authors participated in the conception of the paper. JU drafted the paper. TK, NS, ZI, IK, and MQ participated in drafting some sections of the paper. All the authors reviewed and contributed to revising the paper, and they have seen and approved the final version.
